# Coccygectomy for refractory coccydynia, old-fashioned but effective procedure: A retrospective analysis

**DOI:** 10.1007/s00264-024-06236-y

**Published:** 2024-06-18

**Authors:** Andrea Perna, Andrea Franchini, Luca Macchiarola, Francesco Maruccia, Felice Barletta, Francesco Bosco, Giuseppe Rovere, Franco Lucio Gorgoglione

**Affiliations:** 1 Department of Orthopedics and Trauma Surgery, Fondazione Casa Sollievo Della Sofferenza IRCCS, 71013 San Giovanni Rotondo, Italy; 2 Department of Orthopedics, Università Cattolica del Sacro Cuore, 00168 Rome, Italy; 3 Department of Precision Medicine in Medical, Surgical and Critical Care (Me.Pre.C.C.), University of Palermo, Palermo, Italy; 4 Clinical Science and Translational Medicine, Section of Orthopaedics and Traumatology, University of Rome Tor Vergata, 00133 Rome, Italy

**Keywords:** Coccygeal resection, Coccygectomy, Coccygodynia, Coccyx

## Abstract

**Purpose:**

Coccydynia, characterized by persistent pain in the coccygeal region, significantly impacts patients' quality of life. While various treatment modalities exist, including conservative measures and surgical interventions like coccygectomy, optimal management remains unclear. This retrospective cohort study aimed to compare the clinical outcomes, functional improvements, and quality of life in patients with chronic coccydynia undergoing either infiltrative treatment or coccygectomy.

**Methods:**

Data from patients treated at our institution from January 2018 to December 2022 were analyzed. Participants meeting inclusion criteria were divided into two groups: Group A underwent coccygectomy, while Group B received conservative therapy. Clinical assessments, radiographic evaluations, and patient-reported outcomes were collected preoperatively and at follow-up intervals.

**Results:**

Of the 223 initially examined patients, 55 met inclusion criteria. Group A (n = 21) underwent coccygectomy, while Group B (n = 34) received conservative therapy. Both groups showed significant pain reduction post-intervention, with sustained improvement in Group A. Functional outcomes favoured Group A, with significant improvements in disability and quality of life measures. Complications were minimal, with only one case of superficial wound infection in Group A.

**Conclusion:**

Our findings suggest that coccygectomy provides superior and lasting pain relief, functional improvement, and quality of life improvement compared to conservative therapy. While complications were minimal, further research with larger cohorts is warranted to validate these results and explore long-term outcomes. Despite its historical association with complications, advancements in surgical techniques and perioperative care have led to improved outcomes and reduced complication rates. Thus, coccygectomy should be considered in the treatment algorithm for patients with debilitating coccydynia.

## Introduction

Coccydynia is defined as the presence of intense and persistent pain in the coccygeal region, worsening in the seated and supine lying positions [1]. Several classification attempts have been devised for coccydynia; however, many appear incomplete. [2] A comprehensive classification should not rely solely on the site of pain but should encompass elements of pathoanatomy. Nathan et al. [2] have proposed a comprehensive classification. Coccydynia can be categorized by aetiology into (I) idiopathic or (II) traumatic (with the most common cause being a fall from a height resulting in axial trauma). Other traumatic causes include coccygeal subluxation or fractures occurring during childbirth, prolonged sitting on hard and uncomfortable surfaces, repeated microtrauma. [2] Regarding pathoanatomy, causes of coccydynia may include: (I) degeneration of the sacrococcygeal and intercoccygeal discs and joints; (II) coccyx anatomy and shape or the presence of a bony spicule and coccygeal retroversion; (III) coccyx mobility: immobility, hypermobility, or posterior subluxation; (IV) referred pain: lumbar pathology or arachnoiditis of the sacral nerve roots, spasm of the pelvic floor muscles, and inflammation of the pericoccygeal soft tissues; (V) others: neoplasm, crystal deposits, infections. [2]

Coccydynia significantly impacts patients' quality of life and is often an overlooked cause of unrecognized low back pain [3, 4].

Generally, pain is attributed to hypermobility and subluxation of the coccygeal vertebrae at the sacrococcygeal joint [5]. Various treatment modalities have been proposed, with the majority being conservative. Typically, a donut-shaped cushion is prescribed for seated use to reduce axial load on the coccyx. Various physiotherapeutic treatments, such as shockwave therapy, iontophoresis, and osteopathic interventions like manipulations and transanal repositioning maneuvers, have been described, but there is a lack of consensus in the literature [6].

Some authors argue that a series of cortisone and local anesthetic injections may alleviate pain and improve quality of life. Several minimally invasive pain therapy procedures have also been suggested, such as radiofrequency denervation, paracoccygeal ganglion denervation, or the injection of platelet-rich plasma (PRP), yielding conflicting results. [1] It is estimated that the aforementioned conservative treatments may even yield acceptable clinical outcomes in approximately 90% of patients. [6]

Coccygectomy is reserved for patients experiencing intense pain refractory to conservative therapy, but it seems to be burdened with numerous complications, particularly infections, due to the anatomical proximity to the anal orifice. [1] This study aims to compare clinical and functional outcomes, as well as the quality of life, in patients with chronic coccydynia undergoing either infiltrative treatment or coccygectomy.

## Materials and methods

### Study design and Institutional database

This work represents a retrospective observational, single-centre cohort study on patients with chronic coccydynia, who were assessed and treated at our institution from January 2018 to December 2022. The study was conducted according to Strengthening the reporting of observational studies in epidemiology (STROBE) guidelines.

All enrolled patients in the study provided consent for the collection of scientific data and privacy. The collected data are anonymized to ensure patient privacy and non-identifiability. Given the retrospective design of the study and the examined procedures being a standard of care in our hospital, coupled with the completely anonymous data collection ensuring the privacy of enrolled patients, formal ethical committee approval is deemed unnecessary. The study was conducted in accordance with the 1964 Declaration of Helsinki and its subsequent amendments. From the outpatient visit records of each patient, the following data were extracted: age, gender, comorbidities, Body mass index (BMI), smoking habits, diabetes presence, pain assessment using the Numerical Rating Scale (NRS), functionality assessment through the Short-form 36 (SF36), and the Oswestry disability index (ODI) questionnaire. Additionally, the presence of complications during treatment was recorded for all enrolled patients in the study.

### Participants, Inclusion and exclusion criteria

**Fig. 1:**
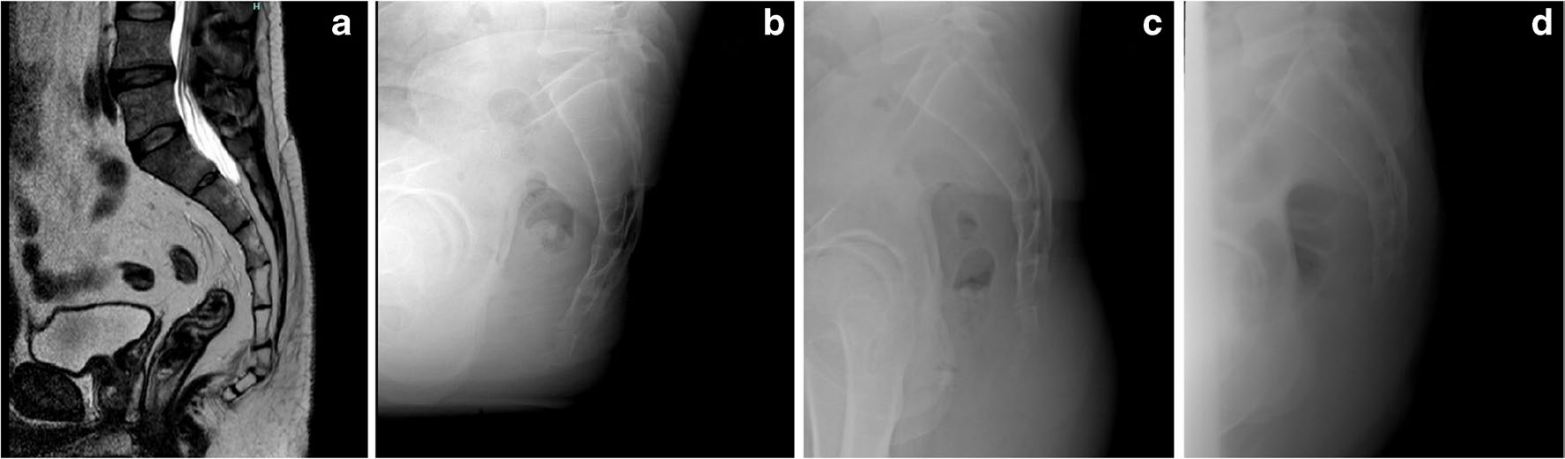
Radiographic images of an illustrative case. **a**. Sagittal cut of a T2-weighted MRI image highlighting the angulation of the last 3 coccygeal segments. **b,c**. Lateral view X-ray images in seated and standing positions illustrating the instability of the last coccygeal segments. **d**. Lateral view X-ray showing the removal of the last coccygeal segments

All patients who accessed our institution due to coccydynia during the reference period were potentially eligible for the study. Inclusion criteria comprised chronic coccydynia persisting for at least 6 months with poor response to pharmacological treatment the presence of a subluxation or hypermobility defined by an angle of at least 25° in lateral view sacro-coccigeal in sitting position X-rays [7] as reported in Fig. 1, age of at least 18 years, a minimum follow-up of two years, and completeness of clinical and radiographic documentation. Exclusion criteria included proctological conditions such as anal fistulas, fissures, haemorrhoids, or pilonidal sinus, diabetes mellitus, immune disorders, tumors, prior spinal surgery, or cortisone allergy or intolerance. To all patients who were ultimately included in the study, a coccygectomy procedure was suggested. However, not all of them accepted; therefore, the patients were divided into two groups based on the chosen treatment. Group A patients who underwent coccygectomy surgery, while Group B, represented the control group treated with a physiotherapy protocol and a series of infiltrations with corticosteroids and local anaesthetic.

### Surgical technique

**Fig. 2:**
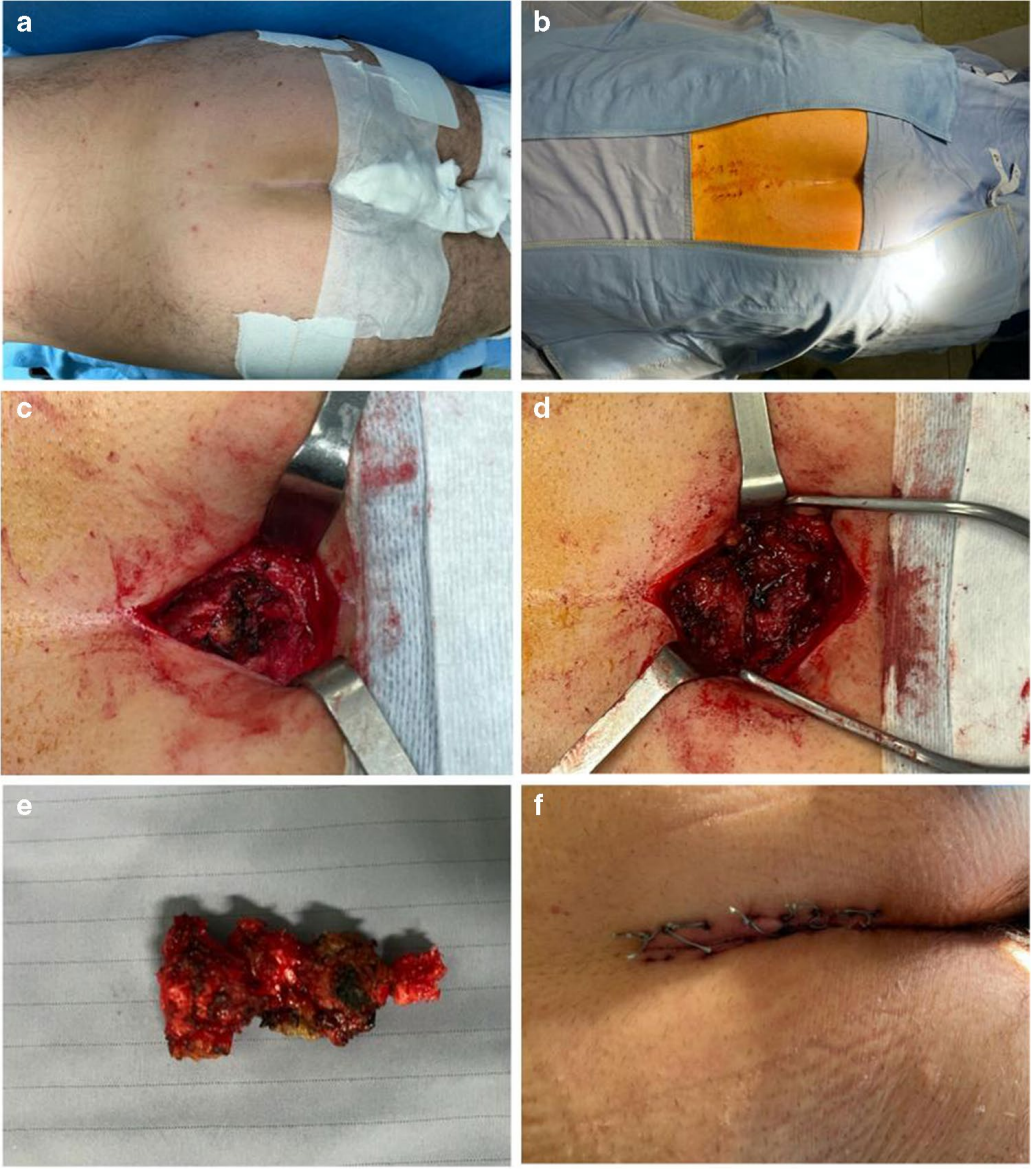
In this series, intraoperative images of an illustrative case are depicted. **a**. Preoperative preparation with gauzes soaked in antiseptic solution at the intergluteal cleft level. **b**. Preoperative sterile field. **c**. Exposure of mobile coccygeal segments. **d**. Surgical field after removal of the last 3 coccygeal segments. **e**. Coccygeal segments removed en bloc. **f**. Closure of the surgical wound with mattress sutures using non-absorbable suture material

All surgical procedures were conducted by a team consisting of two experienced orthopedic surgeons specializing in spinal surgery, using the same technique. Surgically treated patients received preoperative antibiotic prophylaxis intravenously with 2 g ceftriaxone and 400 mg teicoplanin, with the infusion completed approximately 30 min before incision. All operated patients received an evacuative enema the evening before the surgery and an urinary catheter. All patients underwent surgery under general anaesthesia in the prone position on a Wilson frame. Sterile field preparation included placing a gauze soaked in chlorhexidine-based disinfectant at the anal orifice as reported in Fig. 2a. The procedure followed a technique similar to that described by Key in 1937 [8]. A longitudinal incision of 4 cm was made at the midline of the sacrococcygeal region with a slight lateral deviation distally to avoid the intergluteal cleft. Dissection of subcutaneous and pericoccygeal tissues exposed the last coccygeal vertebrae and the sacrococcygeal joint as reported in Fig. 2c. After exposing the mobile coccygeal vertebrae, they were separated from the anterior perirectal tissue and resected following an incision at the sacrococcygeal joint. The residual sacral surface was finished with an osteotome to avoid subcutaneous protrusions. Extreme care was taken to avoid injury to the filum terminale or rectum. Cauterization of pericoccygeal tissues was performed to achieve denervation. Haemostasis was ensured, followed by extensive irrigation, and subsequently, all layers were closed. The skin was sutured with mattress stitches using non-absorbable braided 0 polyethylene terephthalate filament as reported in Fig. 2f. No surgical drains were placed in any patient. After the procedure, a sterile compressive dressing was applied.

### Conservative management protocol

Patients in group B were menaged with the following six month conservative treatment protocol. Throughout this period, they were encouraged to use a donut-shaped cushion when in a sitting position. Initially, they underwent a series of infiltrative therapy with three pericoccygeal injections administered approximately three weeks apart, consisting of Triamcinolone acetonide 40 mg/ml (1 ml) and mepivacaine 30 mg/ml (2 ml). The injections were performed in the pericoccygeal tissues without the aid of radiographic or ultrasound guidance. Subsequently, all patients underwent a physiotherapeutic and instrumental treatment, including three cycles of ten sessions of massages for the coccygeus and levator ani muscles, and three sessions of low-intensity shockwave therapy at approximately 2000 pulses per session, spaced two weeks apart [9].

### Peri and postoperative follow up

All enrolled patients underwent magnetic resonance imaging (MRI) and antero-posterior and lateral-lateral radiographs of the sacro-coccygeal region. Based on the radiographs, patients were classified according to the Postacchini and Massobrio classification [10]. The number of coccygeal segments and the angle between the coccyx and sacrum were also determined. Following surgical intervention, all operated patients received antibiotic therapy with intravenous administration of 2 g ceftriaxone and 400 mg teicoplanin daily for two postoperative days.

Patients and their families were educated on daily wound care, involving iodopovidone-based solutions, and antiseptic soaps were recommended for daily cleansing of intimate areas. The use of a donut-shaped cushion for the subsequent four months during sitting was advised. Additionally, a suitable, low-residue diet was suggested for the first month post-surgery. Stitch removal occurred approximately 15 days after the surgical procedure.

### Clinical evaluation

Patients underwent clinical assessments at one month, six months, one year post-surgery. Additional evaluations were conducted at each follow-up visit, employing the SF36 questionnaire to assess the Physical Component Score (PCS) and Mental Component Score (MCS), [11] the ODI questionnaire [12], and the NRS for sacro-coccygeal pain. Subsequent telephone interviews were conducted in 2023, at least after two years of follow up, during which, in addition to the aforementioned questionnaires, patients were asked to provide feedback on their treatment experience. Patient satisfaction was gauged by having them rate their experience on a numerical scale from 1 to 10. During follow-up visits, any complications and the corresponding treatments were documented.

### Variables

The primary outcomes of this study consist of clinical assessment using the NRS scale and functional evaluation through the SF36 and ODI. Secondary outcomes include the number of complications observed during the follow-up and the overall satisfaction index of patients with the received treatment.

### Statistical analysis

The statistical analysis was performed using dedicated software, PRISM by GraphPad Software. Values are presented as means and percentages, and rounding to one decimal place was applied. The standard deviation (SD) for each calculated value was reported. The Mann–Whitney U-test was employed to analyze independent ordinal variables, while the Wilcoxon Signed-Rank Test was used for the analysis of two dependent ordinal variables. Chi-square test was used for categorical data. The normality of the study population was assessed with the Shapiro–Wilk test. As the population did not exhibit a normal distribution, ANOVA analysis was not suitable and therefore was not conducted. Statistical significance was established for a p-value < 0.05.

## Results

### Patients

Out of 223 retrospectively examined patients, only 68 met the inclusion and exclusion criteria. However, only 55 of them completed the survey proposed in this study and were thus enrolled. Group A comprised 21 patients, while Group B included 34 individuals. The average age of the patients was 41.8 (± 11.4) years, with a female-to-male ratio of 3.2 to 1. Despite not adhering to a normal distribution, the two patient groups exhibited homogeneity in terms of conditions, age, BMI, duration of painful symptoms, and etiology of coccygeal pain. The mean follow-up duration was 32.8 (± 7.2) months. The primary patient characteristics and distinctions between the two study groups are summarized in Table 1.

**Table 1:** Demographic features; p value are between Group A and Group B. a: Wilcoxon Signed-Rank Test; b: Chi-square test; c: Mann Whitney test

Demographics	All patients enrolled	Group A	Group B	p value*
Number of patients	55	21	34	
Age	41.8 y (± 11.4)	40.2 y (± 9.7)	42.2 y (± 12.7)	> 0.05 a
Sex	M:13; F:42	M:5; F16	M:8; F26	
Body mass index (BMI)	28.7 (± 4.4)	27.3 (± 3.1)	29.1 (± 3.5)	> 0.05 a
Depression	14 (25.4%)	6 (28.5%)	8 (23.5%)	> 0.05 b
Fibromyalgia	15 (27.3%)	4 (19%)	11 (32.4%)	> 0.05 b
Rheumatic disease	4 (7.2%)	1 (4.8%)	3 (8.8%)	> 0.05 b
Duration of pain (months)	14 .2 (± 5.3)	16.1 (± 4.6)	13.6 (± 4.9)	> 0.05 c
Smokers	28 (50.9%)	11 (52.4%)	17 (50%)	> 0.05 b
Etiology				
Traumatic	27 (49.1%)	10 (47.6%)	17 (50%)	> 0.05 b
Delivery	15 (27.1%)	6 (28.7%)	9 (26.5%)	> 0.05 b
Hypermobility	10 (18.1%)	4 (19%)	6 (17.6%)	> 0.05 b
Others	3 (5.7%)	1 (4.7%)	2 (5.9%)	> 0.05 b
Number of coccygeal vertebrae				
2	23 (41.6%)	9 (43.2%)	14 (41.2%)	> 0.05 b
3	26 (47%)	10 (47.6%)	16 (47.1%)	> 0.05 b
4	6 (11.4%)	2 (9.2%)	4 (11.7%)	> 0.05 b
Postacchini Massobrio Classification [10]				
1. Curving slightly forward	24 (43.4%)	9 (43.2%)	15 (42.9%)	> 0.05 b
2.Curving strongly, tip straight forward	17 (30.8%)	6 (28.6%	11 (32.4%)	> 0.05 b
3. Angled sharply forward	9 (16.3%)	2 (9.2%)	7 (21.8%)	0.035 b
4.Partly dislocated forward at one of the joints	5 (5.9%)	4 (19%)	1 (2.9%)	0.042 b
Mean Follow Up (months)	32.8 (± 7.2)	34.8 (± 8.1)	31.9 (± 6.5)	> 0.05 c

### Surgical data

The average duration of the surgical procedure was 41 (± 23) minutes. No intraoperative complications were observed in patients belonging to Group A. Blood loss was minimal and, therefore, not quantifiable. All patients were mobilized on the first postoperative day. The urinary catheter was removed on the second postoperative day.

### Clinical and functional results

A significant reduction in NRS was observed in both Group A and Group B between preoperative values and those at one month post-surgery (in Group A, from 9.4 ± 3.1 to 5.2 ± 2.3) or one month after the end of the conservative protocol (in Group B, from 9.1 ± 2.6 to 4.1 ± 3.2). The reduction in pain measured with the NRS scale is sustained over time in Group A (1.8 ± 1.1 after one year post-surgery), whereas this result was not achieved in Group B (7.8 ± 2.3 after one year from the conservative treatment protocol). The differences between the two groups regarding NRS are statistically significant after six months post-surgery or post-conservative protocol, as reported in Table 2. Regarding functional outcomes, ODI and SF-36, Group A showed better values compared to Group B. For ODI, we recorded an improvement in Group A from 41.4 ± 9.8 preoperatively to 24.1 ± 9.8 postoperatively, and this reduction is statistically significant; the improvement continued until the last follow-up to a value of 10.1 ± 5.7. However, for Group B, the slight improvement in ODI was not statistically significant, changing from 40.4 ± 8.9 pre-treatment to 31.2 ± 8.9 at the last follow-up. For SF-36, a statistically significant improvement occurred in Group A starting from six months after the surgery. Such improvement was not observed in Group B. The obtained results are reported in Table 2.

**Table 2:** Main Clinical and functional outcomes

	Pre treatment	1 month	6 months	1y	Last Follow up	P value *,** value compared
NRS Group A	9.4 (± 3.1)*	5.2 (± 2.3)	2.1 (± 1.2)	1.9 (± 0.9)	1.8 (± 1.1)**	0.004 a
NRS Group B	9.1 (± 2.6)*	4.1 (± 3.2)	5.6 (± 2.4)	6.1 (± 3.3)	7.8 (± 2.3)**	> 0.05 a
p Value A *vs* B	> 0.05 a	> 0.05 a	0.035a	0.024 a	0.017 a	
ODI Group A	41.4 (± 9.8)*	36.3 (± 11.2)	24.1 (± 9.8)	12.4 (± 6.2)	10.1 (± 5.7)**	0.012 a
ODI Group B	40.4 (± 8.9)*	33.4 (± 12.3)	35.8 (± 8.8)	32.3 (± 9.2)	31.2 (± 8.9)**	> 0.05 a
p Value A *vs* B	> 0.05 a	> 0.05 a	0.047 a	0.037 a	0.026 a	
SF 36 (MCS) Group A	42.7 (± 9.8)*	54.8 (± 10.2)	62.7 (± 12.3)	72.4 (± 11.2)	73.1 (± 12.7)**	0.003 a
SF 36 (MCS) Group B	43.9 (± 10.1)*	50.2 (± 10.7)	54.2 (± 11.9)	55.1 (± 13.1)	54.9 (± 9.7)	> 0.05 a
P value A *vs* B	> 0.05 a	> 0.05 a	> 0.05 a	0.041 a	0.014 a	
SF 36 (PCS) Group A	35.3 (± 8.2)*	41.6 (± 9.7)	56.1 (± 9.1)	61.3 (± 9.5)	62.5 (± 10.7)**	0.001 a
SF 36 (PCS) Group B	37.4 (± 9.0)*	45.6 (± 8.9)	46.3 (± 12.3)	46.7 (± 10.2)	46.4 (+ /9.3)**	> 0.05 a
P value A *vs* B	> 0.05 a	> 0.05 a	> 0.05 a	0.043 a	0.038 a	

a: Mann Whitney test; MCS: Mental component score; NRS: Numeric rate scale; ODI: Oswestry disability index; PCS: Physical component score; SF 36: Short Form 36;

### Complications

In a single case belonging to Group A, a superficial surgical wound infection with wound dehiscence occurred. The patient responded poorly to specific antibiotic therapy, necessitating a surgical wound revision intervention. Following the revision, the surgical wound is completely healed, and the patient is satisfied with the outcome achieved. No other complications were reported in Group A. Regarding Group B, transient hypertensions occurred in two patients following local infiltration.

## Discussion

Coccygodynia represents a rare but intensely painful condition. Despite its impact on individuals' quality of life, coccydynia remains relatively underexplored in epidemiological studies. The exact incidence and prevalence rates of coccygodynia are not well-defined, but it is generally acknowledged to represent a minority of cases within the spectrum of back pain, accounting for less than 1% of reported cases. However, this condition may be underestimated due to under reporting and misdiagnosis. [13] Coccygodynia is not limited to a specific age group, although it is more commonly observed in individuals over the age of 40 [14, 15]. Nevertheless, it can affect individuals across various age groups, including younger adults and adolescents [15]. Women appear to be affected by coccydynia more frequently than men, as reported by several studies in the literature [9, 14, 15]. Our data confirm what is reported in the literature; indeed, the female-to-male ratio observed in our study is 3.2 to 1. The aetiology of coccydynia is multifactorial, with various factors contributing to its development. Hypermobility and subluxation of the coccyx, often associated with trauma or repetitive strain, are recognized as common culprits. Additionally, anatomical variations in coccyx morphology may predispose individuals to the condition, leading to pain and discomfort in the coccygeal region.Several risk factors have been identified for the development of coccydynia. [16] Increased body mass index (BMI) has been associated with a higher risk of experiencing coccygeal pain, likely due to increased pressure and strain on the coccyx and surrounding structures. [6, 17] Furthermore, women who have undergone two or more vaginal deliveries are at an elevated risk of developing coccydynia, possibly due to the stress placed on the coccyx during childbirth. [16] In accordance with the literature, traumatic aetiology appears to be the most frequent. In this case as well, our case series aligns with the literature, reporting a traumatic aetiology in 49.1% of cases. [16]

A plethora of conservative treatment modalities have been delineated for managing coccydynia, aiming to alleviate pain and improve patients' quality of life. Among these, pharmaceutical interventions reign supreme, with nonsteroidal anti-inflammatory drugs (NSAIDs) and corticosteroids serving as frontline agents. These medications are often complemented by cycles of analgesic and instrumental physiotherapy, targeting pain relief and functional restoration. [18]

In addition to these conventional approaches, more invasive interventions have emerged as potential solutions for refractory cases. Techniques such as local anaesthetic and cortisone infiltrations, Ganglion Impar Blocks (GIB), and Ganglion Impar Radiofrequency Thermocoagulation (RFT) have been proposed, offering targeted pain management when conservative measures fail to yield adequate symptom control. [19, 20]

However, despite the array of therapeutic options available, the efficacy of conservative treatments over the long term remains a subject of scrutiny. While short-term outcomes may show promising symptom reduction, longitudinal studies paint a more nuanced picture. Indeed, research indicates that although symptoms may diminish over time with conservative management, a substantial proportion of patients continue to experience persistent discomfort even at extended follow-up intervals, such as 36 months [6]. However, our data regarding conservative treatment outcomes are much poorer compared to those reported in the literature [6]. This could be attributed to several factors, foremost among them being the administration of infiltrations without radiographic or ultrasound guidance. Blinded infiltrations, indeed, appear to be more imprecise and less effective; it is likely that the conservative treatment protocol was not optimal, thus potentially introducing a bias into the study.

Coccygectomy represents a treatment option for coccydynia, typically considered when conservative treatments or ganglion impar block fail.

Historically, coccygectomy has been described as a procedure associated with a high rate of complications, hence discouraged by some authors [14]. However, in recent decades, numerous studies have reported good clinical outcomes and a reduced rate of complications compared to the past considering coccygectomy a safe procedure[15, 21, 22].

For instance, Sarmast et al. [15] conducted a retrospective study involving 16 consecutive patients who underwent coccygectomy, reporting a substantial decrease in patients' pain levels, as measured by the visual analog scale (VAS), from 9.62 preoperatively to 2.25 postoperatively, with a positive outcome rate of 87.5%. Similarly, Hochgatterer et al. [21] conducted a retrospective study analyzing data from patients who underwent coccygectomy, with a follow-up period extending up to 29 years. They observed a significant reduction in pain levels, as indicated by VAS scores decreasing from 6.37 to 0.68, accompanied by few reported complications. Additionally, the authors argued that the classification of the coccyx according to Postacchini did not significantly influence the final outcome. Milosevic et al. [22] reported findings from 112 cases of coccygectomy with a follow-up period of at least one year. They noted significant improvements in various outcome measures, including VAS (from 70.99 to 35.34), Oswestry Disability Index (ODI, from 31.84 to 18.00), EuroQol five-dimensional questionnaire (EQ-5D, from 0.52 to 0.75), and SF-36 Physical Component Summary (PCS, from 38.17 to 44.74). Moreover, over 70% of the patients in this study reported favorable outcomes following treatment. Consistent with the existing Literature, our study demonstrated a significant improvement in pain, as measured by the Numerical Rating Scale (NRS), and notable enhancements in ODI and SF-36 scores, both in PCS and MCS, among patients treated surgically compared to those managed conservatively.

Superficial and deep wound infections represent the most common complications in coccygeal surgery, occurring in up to 22% of cases, while the reported complication rate in the Literature generally ranges from 10 to 50% [23]. However, in our study, infectious complications remained markedly lower, with an average rate of 4.7% (in only one patient). We attribute this lower incidence to the utilization of dual antibiotic prophylaxis and the administration of intravenous antibiotics for at least 48 h post-procedure. Our assertion is supported by the study conducted by Doursounian and colleagues [24], who reported a reduction to 0% in infection rates among patients treated with coccygectomy, attributed to the implementation of novel wound management techniques and aggressive antibiotic prophylaxis.

### Limitations

This study is not without limitations. As a retrospective study, it is inherently susceptible to biases associated with this design. Additionally, the relatively low number of patients enrolled is attributed to the rarity of the condition under investigation. A potential bias in the study stems from a suboptimal conservative treatment protocol, resulting in poorer outcomes observed in the control group. Consequently, further research with a more robust study design is warranted to corroborate our findings.

## Conclusion

Coccygodynia is a condition that poses significant challenges and profoundly affects the quality of life of patients. Despite the availability of various conservative treatments, there remains a subset of patients who do not respond well to these methods, prompting the need for more invasive interventions. Our findings indicate that coccygectomy provides substantial and long-lasting pain relief, enhances functionality, and improves overall quality of life compared to conservative management. Additionally, the occurrence of complications, particularly infectious ones, was remarkably low among patients who underwent surgery, highlighting the safety and effectiveness of coccygectomy when performed by skilled surgeons.

## Data Availability

Data available on request from the authors.

## Declarations

The collected data are anonymized to ensure patient privacy and non-identifiability. Given the retrospective design of the study and the examined procedures being a standard of care in our hospital, coupled with the completely anonymous data collection ensuring the privacy of enrolled patients, formal ethical committee approval is deemed unnecessary. The study was conducted in accordance with the 1964 Declaration of Helsinki and its subsequent amendments.

## References

[1] Kara D, Pulatkan A, Ucan V, Orujov S, Elmadag M (2023) Traumatic coccydynia patients benefit from coccygectomy more than patients undergoing coccygectomy for non-traumatic causes. J Orthop Surg Res. 18:802. Published 2023 Oct 27. 10.1186/s13018-023-04098-5 10.1186/s13018-023-04098-5 PMC10605957 37891674

[2] Nathan ST, Fisher BE, Roberts CS (2010). Coccydynia: a review of pathoanatomy, aetiology, treatment and outcome. J Bone Joint Surg Br.

[3] Perna A, Ricciardi L, Barone G, Tamburrelli FC, Proietti L, Pola E (2018). Medical management of acute non-specific low back pain: comparison of different medical treatments, one center's retrospective analysis. J Biol Regul Homeost Agents.

[4] Meluzio MC, Smakaj A, Perna A (2022). Epidemiology, diagnosis and management of Baastrup's disease: a systematic review. J Neurosurg Sci.

[5] Maigne JY, Tamalet B (1996). Standardized radiologic protocol for the study of common coccygodynia and characteristics of the lesions observed in the sitting position. Clinical elements differentiating luxation, hypermobility, and normal mobility. Spine (Phila Pa 1976).

[6] Charrière S, Maigne JY, Couzi E (2021). Conservative treatment for chronic coccydynia: a 36-month prospective observational study of 115 patients. Eur Spine J.

[7] Maigne JY, Lagauche D, Doursounian L (2008). Instability of the coccyx in coccydynia. J Bone Jt Surg Br.

[8] Key J. (1937) Operative treatment of coccygodynia. JBJS. 19:759–64

[9] Izci EK, Keskin F (2023). Coccygectomy for coccygodynia: A single-center experience. Medicine (Baltimore).

[10] Postacchini F, Massobrio M (1983). Idiopathic coccygodynia. Analysis of fifty-one operative cases and a radiographic study of the normal coccyx. J Bone Joint Surg Am.

[11] Ware JE, Sherbourne CD (1992). The MOS 36-item short-form health survey (SF-36) I Conceptual framework and item selection. Med Care.

[12] Monticone M, Baiardi P, Ferrari S (2009). Development of the Italian version of the Oswestry Disability Index (ODI-I): A cross-cultural adaptation, reliability, and validity study. Spine (Phila Pa 1976).

[13] Kleimeyer JP, Wood KB, Lønne G (2017). Surgery for refractory coccygodynia. Spine.

[14] Soliman AY, Abou El-Nagaa BF (2020). Coccygectomy for refractory coccydynia: a single-center experience. Interdiscip Neurosurg.

[15] Sarmast A, Kirmani A, Bhat A (2018). Coccygectomy for coccygodynia: a single center experience over 5 years. Asian J Neurosurg.

[16] Almetaher HA, Mansour MA, Shehata MA (2021). Coccygectomy for chronic refractory coccygodynia in pediatric and adolescent patients. J Indian Assoc Pediatr Surg.

[17] Maigne JY, Doursounian L, Chatellier G (2000). (2000) Causes and mechanisms of common coccydynia: role of body mass index and coccygeal trauma. Spine.

[18] Sukun A, Cankurtaran T, Agildere M, Weber MA (2023) Imaging findings and treatment in coccydynia - update of the recent study findings. Bildgebende Befunde und Behandlung bei Kokzygodynie – Aktualisierung gemäß der jüngsten Studienergebnisse. Rofo. Published online November 9, 10.1055/a-2185-8585 10.1055/a-2185-8585 37944937

[19] Ghai A, Jangra P, Wadhera S (2019). A prospective study to evaluate the efficacy of ultrasound-guided ganglion impar block in patients with chronic perineal pain. Saudi J Anaesth.

[20] Sencan S, Kenis-Coskun O, Demir FGU (2018). Ganglion Impar block improves neuropathic pain in coccygodynia: A preliminary report. Neurol Neurochir Pol.

[21] Hochgatterer R, Gahleitner M, Allerstorfer J (2021). Coccygectomy for coccygodynia: a cohort study with a long-term follow-up of up to 29 years. Eur Spine J.

[22] Milosevic S, Andersen G, Jensen MM. et al (2021) The efficacy of coccygectomy in patients with persistent coccydynia. Bone Joint J 103-b: 542–546 10.1302/0301-620x.103b3.Bjj-2020-1045.R2. 10.1302/0301-620X.103B3.BJJ-2020-1045.R2 33641429

[23] Karadimas EJ, Trypsiannis G, Giannoudis PV (2011). Surgical treatment of coccygodynia: an analytic review of the literature. Eur Spine J.

[24] Doursounian L, Maigne JY, Cherrier B, Pacanowski J (2011). Prevention of post-coccygectomy infection in a series of 136 coccygectomies. Int Orthop.

